# Comment on: “Gout/hyperuricemia reduces the risk of Alzheimer's disease: A meta‐analysis based on latest evidence”

**DOI:** 10.1002/brb3.3424

**Published:** 2024-02-12

**Authors:** Zain Ali Nadeem, Abdulqadir J. Nashwan

**Affiliations:** ^1^ Department of Medicine Allama Iqbal Medical College Lahore Pakistan; ^2^ Hamad Medical Corporation Doha Qatar

**Keywords:** alzheimer's disease, gout, hyperuricemia

1

Dear Editor,

We read with great interest the recent systematic review and meta‐analysis by Wang et al. (2023), which explored the association of hyperuricemia and gout with Alzheimer's disease. The authors observed a negative correlation between the two conditions detected by a hazard ratio of 0.69 (95% confidence interval 0.66–0.72). The authors concluded that there was a moderate risk of heterogeneity (*I* ^2^ = 93%), which they attributed to regional differences.

In an otherwise excellent paper, we find two methodological inaccuracies that might have overestimated the effect. First, the authors used a fixed‐effect model for their meta‐analysis. The fixed‐effect model ignores any heterogeneity that may be present and gives a more precise effect estimate, but it may not be generalizable. In cases where considerable heterogeneity is present, the random effects model can be used. This model takes into account the different populations on which the individual studies were conducted (Deeks et al., 2019). Second, the authors used funnel plots, Begg's test, and Egger's test to assess the risk of publication bias. As a rule of thumb, the Cochrane Handbook for Systematic Reviews of Interventions recommends against using tests for funnel plot asymmetry when the number of included studies is less than 10 (Page et al., 2019). Additionally, visual inspection of funnel plot asymmetry is quite subjective and prone to errors (Terrin et al., 2005). The Doi plot and the Luis Furuya–Kanamori index are more sensitive to publication bias with less than 10 included studies (Furuya‐Kanamori et al., 2018) and do not suffer from the difficulty in interpretation as do funnel plots.

We commend the authors for searching both Medline and Embase, as recommended by the Cochrane Handbook for Systematic Reviews of Interventions (Lefebvre et al., 2019). We urge Wang and colleagues to assess the impact of using a fixed‐effect model—despite attributing the heterogeneity to the country of origin—discuss its implications on the generalizability of their results, and reassess publication bias using the more sensitive Doi plots.

## AUTHOR CONTRIBUTIONS

**Zain Ali Nadeem**: Writing—original draft; writing—review and editing. **Abdulqadir J. Nashwan**: Writing—review and editing.

## CONFLICT OF INTEREST STATEMENT

The authors declare no conflicts of interest.

### PEER REVIEW

The peer review history for this article is available at https://publons.com/publon/10.1002/brb3.3424.

## Data Availability

Data sharing is not applicable to this article as no datasets were generated or analyzed during the current study.
